# Supplementary material for: Mesophiles vs. Thermophiles: Untangling the Hot Mess of Intrinsically Disordered Proteins and Growth Temperature of Bacteria
Source: Int J Mol Sci. 2024 Feb 7;25(4):2000. doi: 10.3390/ijms25042000 (PMC10889376; doi:10.3390/ijms25042000)
Supplement: Supplementary file 1 [file ijms-25-02000-s001.zip › ijms-2850779-supplementary.pdf]

Supplementary materials

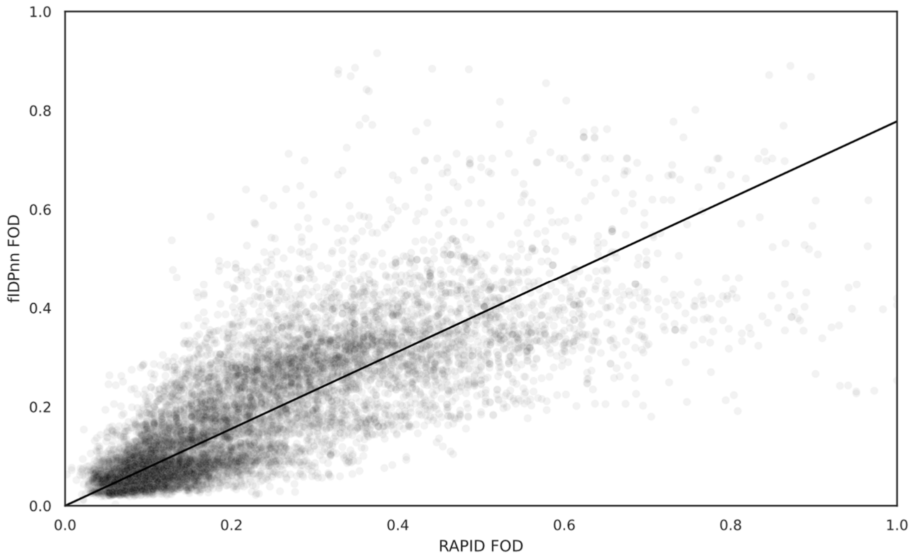

Figure S1. Scatter plot of FOD contrast / OGT contrast relationship.

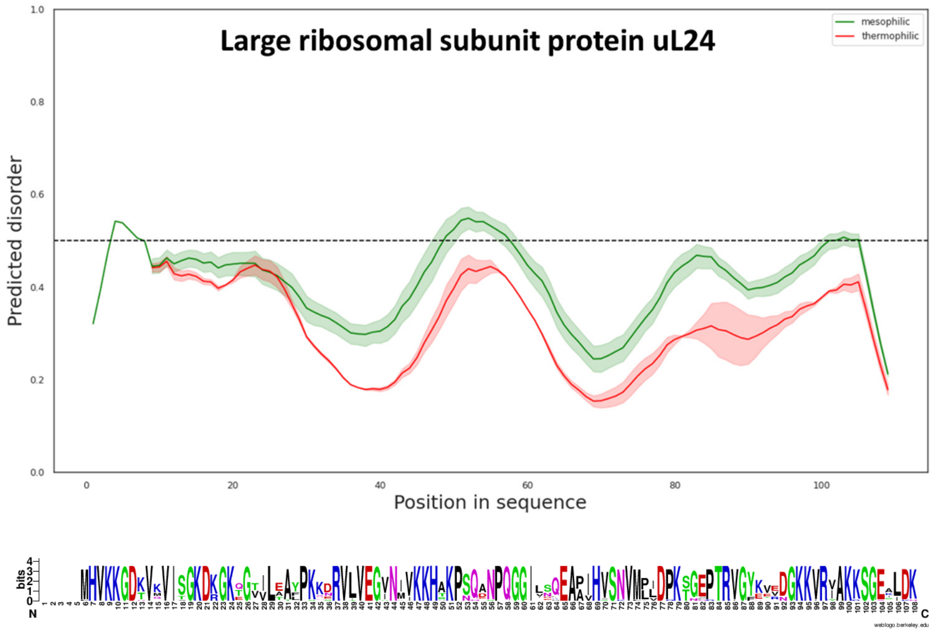

Figure S2. Aligned disorder (uL24) and corresponding WebLogo.

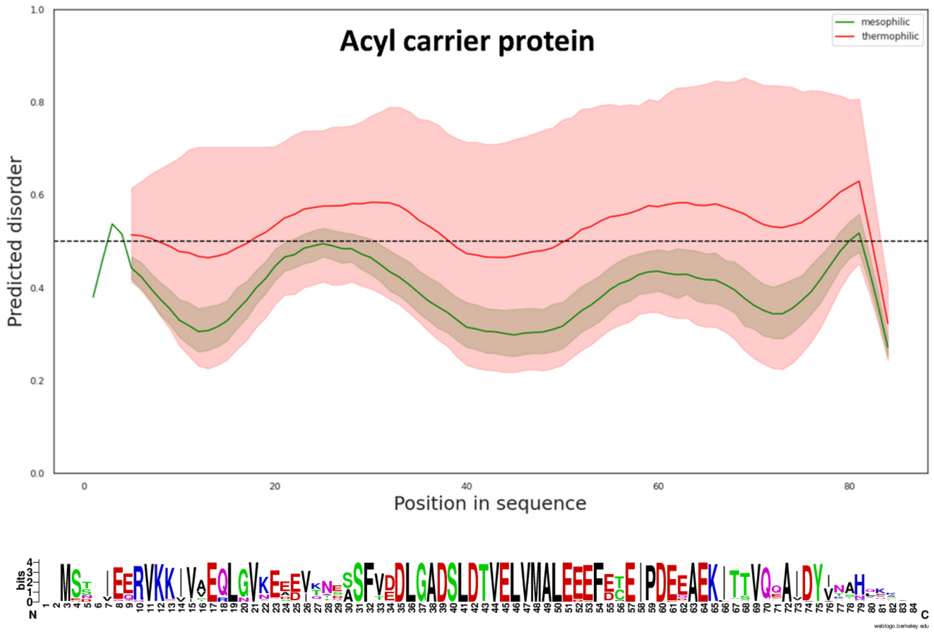

Figure S3. Aligned disorder (Acyl carrier protein) and corresponding WebLogo.

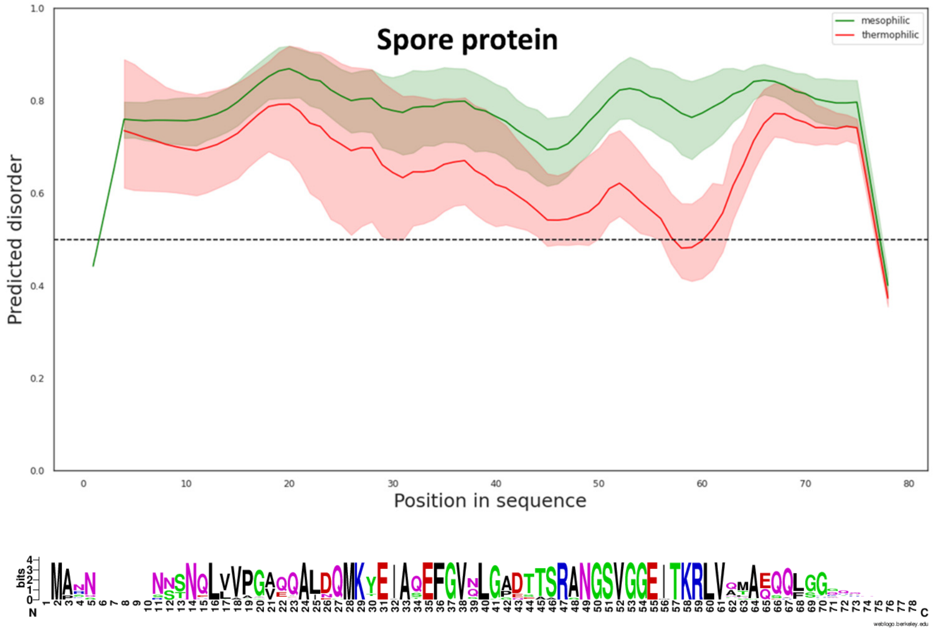

Figure S4. Aligned disorder (Spore protein) and corresponding WebLogo.

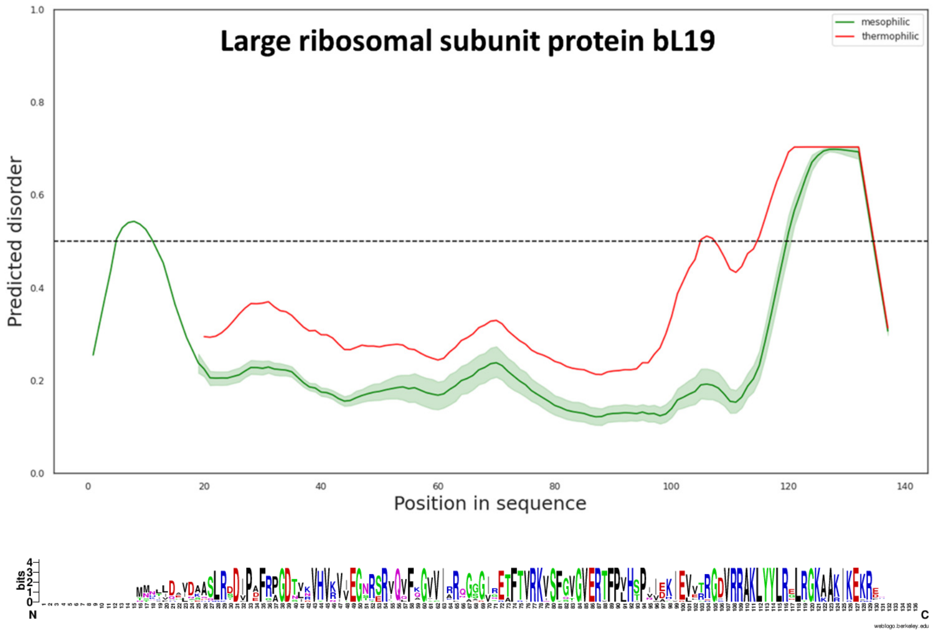

Figure S5. Aligned disorder (bL19) and corresponding WebLogo.

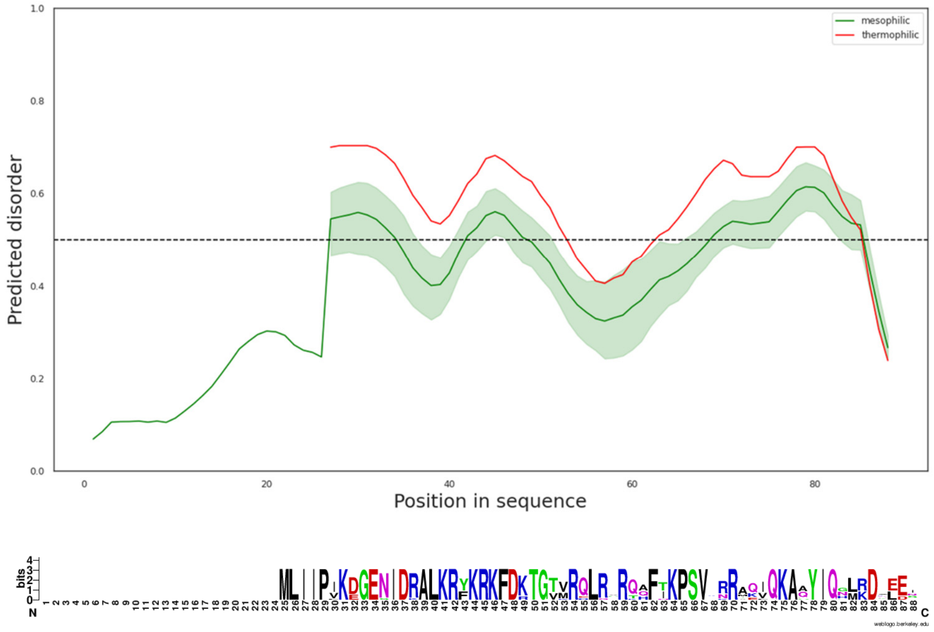

Figure S6. Aligned disorder (bS21) and corresponding WebLogo.

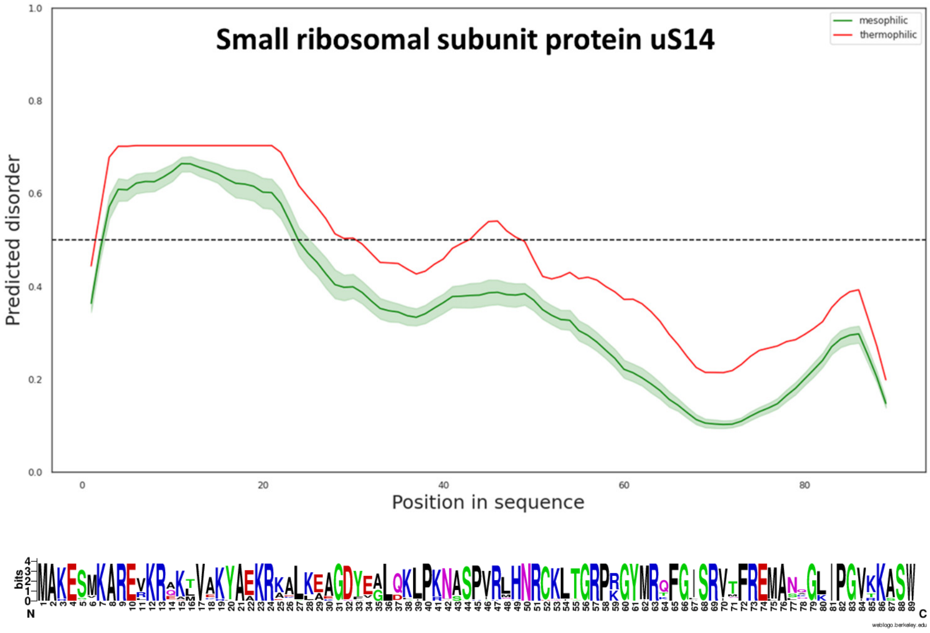

Figure S7. Aligned disorder (uS14) and corresponding WebLogo.

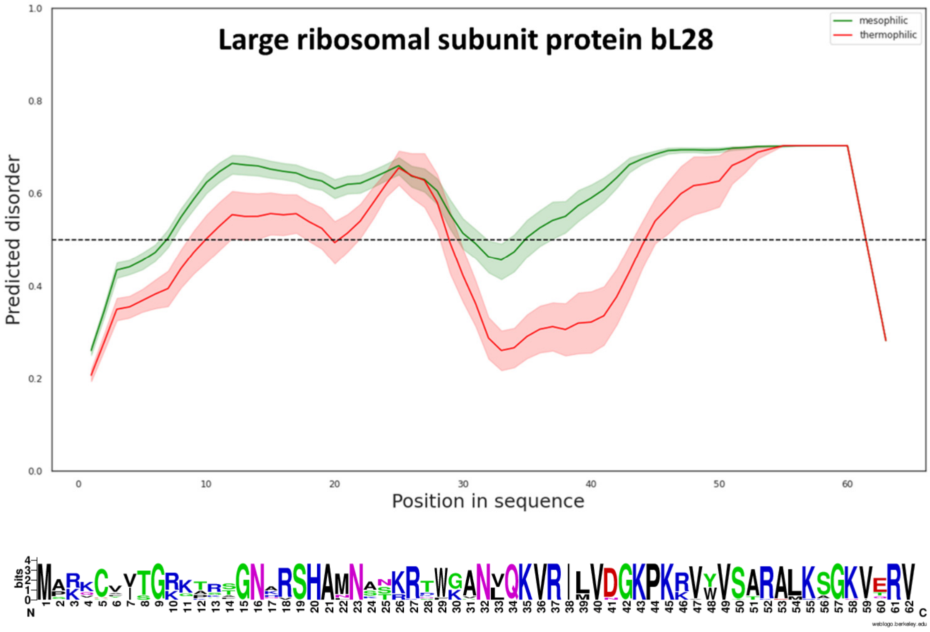

Figure S8. Aligned disorder (bL28) and corresponding WebLogo.

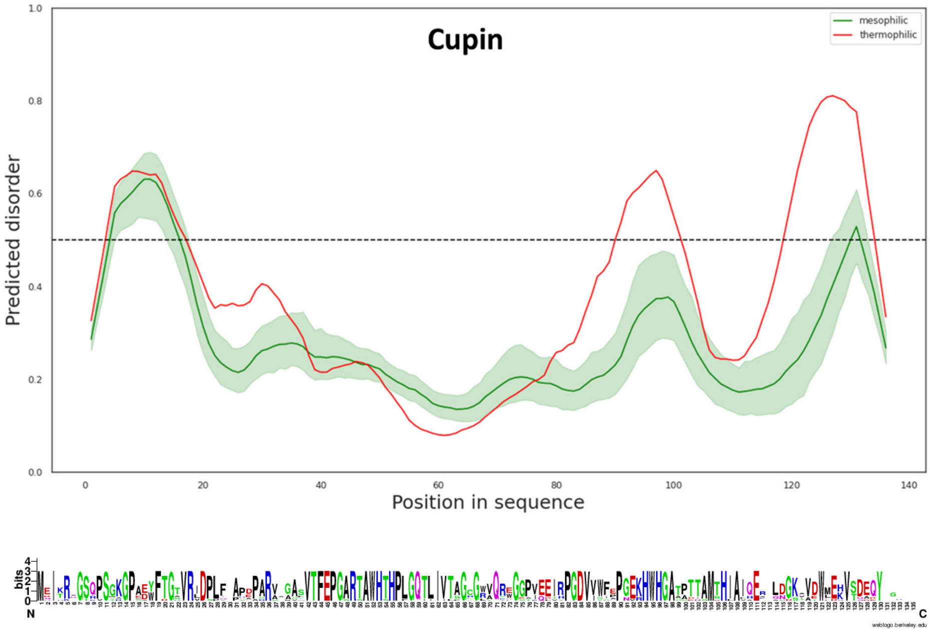

Figure S9. Aligned disorder (Cupin) and corresponding WebLogo.

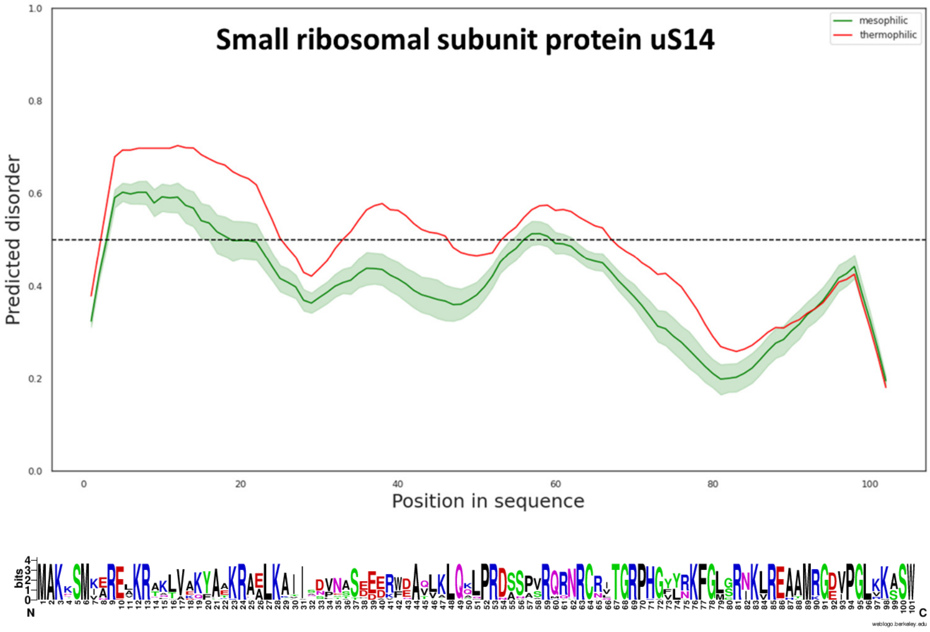

Figure S10. Aligned disorder (uS14) and corresponding WebLogo.

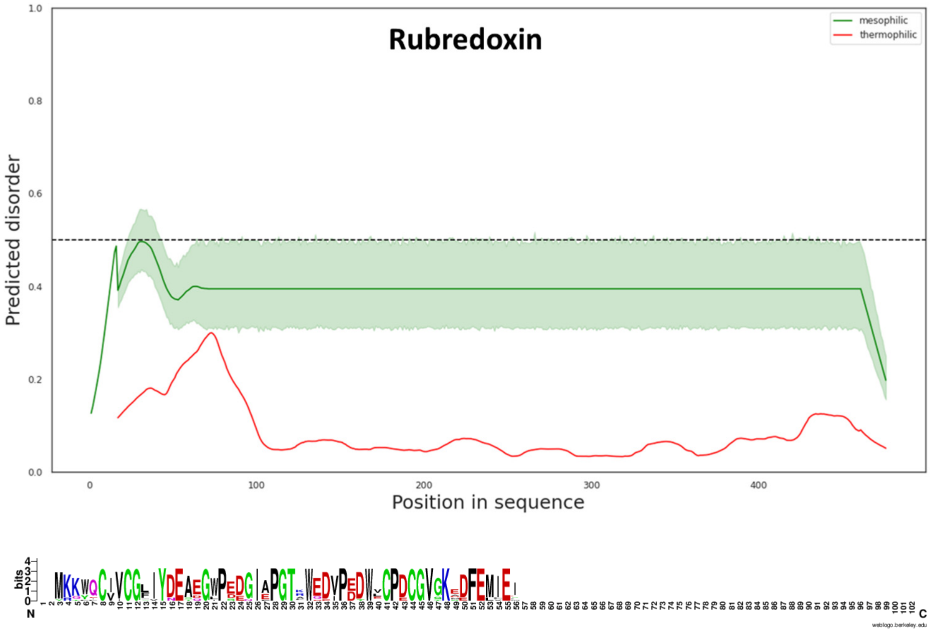

Figure S11. Aligned disorder (Rubredoxin) and corresponding WebLogo. The WebLogo has been limited to 100 first positions because of truncation.
